# The Regulation of Troponins I, C and ANP by GATA4 and Nkx2-5 in Heart of Hibernating Thirteen-Lined Ground Squirrels, *Ictidomys tridecemlineatus*

**DOI:** 10.1371/journal.pone.0117747

**Published:** 2015-02-13

**Authors:** Bryan E. Luu, Shannon N. Tessier, Dianna L Duford, Kenneth B. Storey

**Affiliations:** Institute of Biochemistry & Department of Biology, Carleton University, 1125 Colonel By Drive, Ottawa, Ontario, K1S 5B6, Canada; Universidade de Brasília, BRAZIL

## Abstract

Hibernation is an adaptive strategy used by various mammals to survive the winter under situations of low ambient temperatures and limited or no food availability. The heart of hibernating thirteen-lined ground squirrels (*Ictidomys tridecemlineatus*) has the remarkable ability to descend to low, near 0°C temperatures without falling into cardiac arrest. We hypothesized that the transcription factors GATA4 and Nkx2-5 may play a role in cardioprotection by facilitating the expression of key downstream targets such as troponin I, troponin C, and ANP (atrial natriuretic peptide). This study measured relative changes in transcript levels, protein levels, protein post-translational modifications, and transcription factor binding over six stages: euthermic control (EC), entrance into torpor (EN), early torpor (ET), late torpor (LT), early arousal (EA), and interbout arousal (IA). We found differential regulation of GATA4 whereby transcript/protein expression, post-translational modification (phosphorylation of serine 261), and DNA binding were enhanced during the transitory phases (entrance and arousal) of hibernation. Activation of GATA4 was paired with increases in cardiac troponin I, troponin C and ANP protein levels during entrance, while increases in p-GATA4 DNA binding during early arousal was paired with decreases in troponin I and no changes in troponin C and ANP protein levels. Unlike its binding partner, the relative mRNA/protein expression and DNA binding of Nkx2-5 did not change during hibernation. This suggests that either Nkx2-5 does not play a substantial role or other regulatory mechanisms not presently studied (e.g. posttranslational modifications) are important during hibernation. The data suggest a significant role for GATA4-mediated gene transcription in the differential regulation of genes which aid cardiac-specific challenges associated with torpor-arousal.

## Introduction

Through evolution, animals have developed mechanisms to cope with environmental stressors (e.g. heat, cold, drought, anoxia, lack of food) encountered in their natural habitats. One well-known mammalian survival response is hibernation that allows animals to survive the cold winter months when there is little or no access to food. By abandoning homeothermy, strongly suppressing metabolic rate, and sustaining only processes vital to survival, many small mammals can survive the whole winter using only endogenous body fuel reserves (mainly lipids) to generate energy. During the hibernation season, animals transition through prolonged periods of torpor which are interrupted by brief periods of arousal. During torpor, basal metabolic rate may be depressed by 96–98% compared to euthermia, and core body temperature (T_b_) falls to near ambient (often as low as 0–5°C) [1–5]. For overwintering ground squirrels, studies have shown that the use of hibernation can conserve up to 88% of the energy that would otherwise be needed to remain euthermic over the winter [1]. Ground squirrels prepare for hibernation in autumn by entering a phase of hyperphagia with documented weight gains of up to 40%, prior to switching their metabolism towards a preference for the oxidation of body lipid depots over the long winter months without food [6–7]. Additionally, periods of deep torpor are characterized by reduced organ perfusion (<10% of euthermia), respiration rates (~2.5% of euthermia) and neuronal firing [5,7,8]. The present study analyzes the thirteen-lined ground squirrel (*Spermophilus tridecemlineatus*) to study how a mammalian heart adapts to the stresses encountered during cycles of torpor-arousal.

In order for a mammalian heart to survive under the low temperature and reduced perfusion conditions (which would be normally considered ischemic) of hibernation, heart metabolism is biochemically suppressed in order to restore balance between ATP supply and demand [2,9]. On the physiological level, ground squirrel hearts show a strong reduction in heart rate from 350–400 bpm to 5–10 bpm in torpor, and an increase in contractile strength to deal with colder and more viscous blood [13]. Hibernator hearts also exhibit tolerance of near 0°C temperatures whereas hearts from non-hibernating mammals typically show hypothermia-induced cardiac arrest at about 20–25°C [10,11]. These taxing conditions on the hibernator heart result in cardiac hypertrophy, with characteristics including remodelling of structural proteins and differential regulation of genes/proteins [5,12–13]. Although stressed human hearts can also undergo beneficial cardiac hypertrophy, such changes may transition over time, potentially resulting in cardiac malfunction and heart failure [14–17]. The changes experienced by the heart during progression to pathological hypertrophy include hardening of atrial walls, decreases in blood diastolic volume, and decreases in perfusion efficiency [15]. Although reversible cardiac hypertrophy is observed in the hearts of hibernators, the molecular mechanisms by which this occurs are only beginning to be elucidated [18–22]. The present study focuses on GATA4 and Nkx2–5 transcription factors and downstream genes which have been shown to play a role in attenuating damage caused by cardiac hypertrophy or cytotoxic stress.

Genomic studies have been focusing on elucidating key transcription factors involved in the development of cardiomyocytes [23]. Among these are GATA4, Nkx2–5, and MEF2 that play roles in gene expression, histone modifications, and microRNA regulation during heart development [24]. We became interested in MEF2 involvement in myocyte responses to hibernation and have studied its role in both heart and skeletal muscle of thirteen-lined ground squirrels [22,25]. Many studies have shown that GATA4 and Nkx2–5 are central players in heart metabolism, with GATA4 playing cardioprotective roles in the heart via various mechanisms [26–27]. For example, evidence suggests that GATA4 is essential for mediating erythropoietin-stimulated mechanisms that attenuate myocardial damage due to ischemia/reperfusion injury [26]. GATA4 also promotes cardiomyocyte survival by preventing apoptosis and regulating pathways involved in oxidative stress [27] whereas mutations in GATA4 and Nkx2–5 have been implicated in heart disease [28–29], demonstrating the importance of these transcription factors in normal cardiac function. Post-translational modifications on key residues have been shown to regulate GATA4 activity. The cAMP/PKA signaling pathway can phosphorylate murine GATA4 on Ser261 to increase promoter affinity [30]. Phosphorylation of this residue is also required for recruitment of CREB-binding protein coactivator which facilitates expression of genes [30]. Importantly, Ser261 phosphorylation of GATA4 in rat cardiomyocytes by extracellular signal-related kinases results in acetylation, enhanced stability, association with p300, and cell hypertrophy [31]. Studies have also demonstrated extensive GATA4 / Nkx2–5 crosstalk and dependence. For example, Nkx2–5 regulates atrial natriuretic peptide (ANP) expression, but this cannot occur in the absence of GATA4 [32–38]. Therefore, we became interested in studying these two transcription factors in hopes of shedding light on their roles in hibernating thirteen-lined ground squirrels.

GATA4 and Nkx2–5 transcription factors are known to primarily regulate subunits of the troponin complex (e.g. troponin C, troponin I), ANP, and others in the heart. The troponin complex consists of three different subunits—troponin C, troponin I, and troponin T. Troponin T is a tropomyosin-binding protein which regulates the interaction of the troponin complex with thin filaments. Initiated by action potentials, calcium is released from the sarcoplamic reticulum, binds to troponin C which releases the inhibitory effect of troponin I on acto-myosin ATPase’s and, therefore, plays the main role in Ca^2+^ dependent regulation of muscle contraction. Finally, tropomyosin is released from actin sites for the binding of myosin cross-bridges. While members of the troponin complex regulate heart contraction, they are also used in clinical studies as markers of cardiac stress and damage [39–43].

Studies have shown that the force development of cardiac muscle was significantly greater in hibernating ground squirrels as compared with active animals and heart rate slows prior to any decline in body temperature during entrance into hibernation [44], suggesting that temperature alone cannot explain differences in cardiac contraction. Consequently, we hypothesized that the troponin complex may be differentially regulated in the heart of hibernating squirrels and this may represent a molecular basis to explain changes in heart contraction strength which cannot be explained by temperature effects. In addition, ANP is an anti-hypertensive peptide produced by the heart that has an important role in the regulation of body fluid homeostasis and systemic blood pressure [45–47]. While the local actions of ANP on the heart itself are incompletely understood, studies have shown that it plays an antagonistic role in the development of cardiac myocyte hypertrophy [48]. As such, we wondered if ANP would be differentially regulated in order to re-establish homeostasis as a result of changes in heart rate and blood pressure or be responsive to hypertrophic myocytes.

We sought to identify roles for the transcription factors GATA4 and Nkx2–5, and their downstream targets troponin C, troponin I, and ANP over the course of the torpor-arousal cycle in the heart of thirteen-lined ground squirrels. To do this, we analyzed changes in transcript levels with PCR, quantified relative protein levels and phosphorylation states via immunoblotting, and utilized a modified ELISA assay to measure changes in transcription factor binding to an oligonucleotide containing GATA4 or Nkx2–5 response elements. Differential regulation at crucial time points over the torpor-arousal cycle (entrance and early arousal) outlined in this study suggested that GATA4 and its downstream targets (troponin C, troponin I, and ANP) may play important roles in the hibernator heart.

## Materials and Methods

### Animals Ethics Statement and Experimental Conditions

Thirteen-lined ground squirrels (*Ictidomys tridecemlineatus*), which weighed 150–300g, were wild captured by United States Department of Agriculture (USDA) licensed trappers (TLS Research, Bloomingdale, IL) annually in August. This study did not involve endangered or protected species. Male and female ground squirrels were used equally in the study as a whole with a mixture of genders in each experimental condition and all animals were between 1–3 years of age, although the exact age of the animals is unknown since animals were wild captured. Animals were then transferred to the Animal Hibernation Facility of the National Institute of Neurological Disorders and Stroke (NINDS, Bethesda, MD), as previously described [49]. All animal experiments (including euthanasia) were conducted by the laboratory of Dr. J.M. Hallenbeck prior to shipment to Carleton University (Ottawa, Canada) and, as a result, no live animal studies were performed in Canada. Samples of excised frozen tissues were shipped to Carleton University on dry ice and stored at -80°C before use. The highest standards in ethics and transparency that are applied in North America were used for all experiments. This study, including animal housing and experimental protocols, were approved by the NINDS institutional animal care and use committee (IACUC; Permit Number ASP 1223–05). All surgery was performed under anesthesia and all efforts were made to minimize suffering. Directly after capture, ground squirrels were quarantined in order to ensure they did not contain infectious agents and were treated with 0.5 mL/squirrel of Profender to kill any parasites and prevent disease (3 mg/kg emodepside + 12 mg/kg praziquantel). Each animal was briefly anesthetized with 5% isofluorane and injected subcutaneously in the intrascapular area with a sterile programmable temperature transponder (IPTT-300, Bio medic Data Systems) to allow monitoring of T_b_ during hibernation. To collect tissues, ground squirrels were first anesthetized with 5% isofluorane, the rectal temperature was measured (to verify the temperature transponder), and decapitated rapidly. The tissue samples were quickly rinsed with ice-cold phosphate-buffered saline and frozen instantly in 2-methylbutane chilled with dry ice and then stored in a -80°C freezer until use.

Ground squirrels were held individually in shoebox cages with a constant ambient temperature of 21°C under a 12 h light: 12 h dark cycle until used for experimental purposes. Animals were fed a standard rodent mix and water *ad libitum* until body lipid stores were sufficient to enter hibernation. To facilitate a natural transition into torpor, animals were transferred to an environmental chamber at 5°C at the end of October. So as not to disturb torpid squirrels, a red safe light (3–5 lux) was used when entering the chamber and a heavy dark curtain was used to shield the shelves containing the cages and block the light and sound resulting from opening and closing the door to the environmental chamber. Once a squirrel entered torpor, a small amount of saw dust was dropped on the back of the animal to ensure it exhibited minimal movement during torpor. T_b_, time elapsed, and respiration rates were recorded and used to determine the various stages of the torpor-arousal cycle and when animals would be sampled. All animals, including euthermic controls, had been through several torpor-arousal bouts prior to sampling. Four different animals for each of the six experimental stages were sampled for the present study and all experimental procedures were performed in the laboratory. EC designates euthermic in cold room; euthermic squirrels were capable of entering torpor, but had not for over the previous 72 h, and maintained a stable T_b_ of approximately 37°C. Euthermic controls were capable of entering torpor but had not re-entered hibernation in the past 72 hours. These euthermic animals display the slow-wave sleep characteristic of all sampling animals and were chosen as the reference group to eliminate compounding variables of environmental light, temperature, feeding as well as time/season. EN designates entrance into hibernation; the entrance phase of the torpor-hibernation cycle is characterized by decreasing T_b_ and animals were sampled when T_b_ = 18–31°C. ET designates early torpor; ground squirrels had entered torpor with a constant T_b_ of 5–8°C for ~24 h. LT designates late torpor; animals maintained a T_b_ of 5–8°C for at least 5 days. EA designates early arousal after LT; animals were characterized by a post-torpor increase in T_b_ to 9–12°C, and increased respiration to at least 60 breaths/min. IA designates interbout arousal; animals aroused from LT and had re-established a T_b_ of ~37°C for ~18 h.

### Isolation of Total RNA and cDNA Synthesis

Total RNA was extracted from the hearts of 4 different animals for each of the six experimental stages sampled. Samples of ~100 mg of frozen heart were mixed with1 mL of Trizol reagent (Invitrogen) and processed according to manufacturer’s instructions, as previously described [25]. Purity of RNA was assessed by a 260/280 nm absorbance ratio of 1.8–2.0 and quality was further examined by native agarose gel electrophoresis stained with ethidium bromide. RNA sample concentrations were standardized to 1 μg/μl by adding diethylpyrocarbonate (DEPC)-treated water. For cDNA synthesis, aliquots of 3 μg of total RNA were combined with 7 μl of DEPC-treated water and 1 μl of oligo-dT (Sigma Genosys; 200 ng/μl). Samples were incubated at 65°C in a BioRad iCycler for 5 min, then chilled on ice. To each sample, 4 μL 5X first strand buffer, 2 μL 10 mM dithiothreitol (DTT), 1 μL dNTP (10 mM), and 1 μL Superscript II reverse transcriptase (all Invitrogen) were added. Samples were incubated at 42°C for 1 h and before returning to 4°C. Serial dilutions (10^-1^, 10^-2^, 10^-3^) of the cDNA were made with DEPC-treated water.

### Primer Design

Primers were designed from the consensus sequences of genes from four other mammals (mouse, rat, cow, human) retrieved from the NCBI database. Sequence alignments and identification of conserved regions were done using Geneious (Biomatters, 2009), primers were designed using Primer Designer (v.3.0; Scientific and Educational Software), and primers were synthesized by Sigma Genosys. Primers used were:

1. *gata4* forward 5′-TCCTGTGCCAACTGCCAGAC-3′ and reverse 5′-GTCCCCRTGACTGTCAGCCA-3′,
2. *nkx2–5* forward 5′-CTCACGTCCACGCAGGTCAA-3′ and reverse 5′-TTCCCTACCAGGCTCGGATG-3′,
3. *troponin C* forward 5′-GAGGATGGCTGCATCAGCAC-3′ and reverse 5′-TGTCTCCGTCCTTCATGAGC-3′,
4. *troponin I* forward 5′-TCTAAGATCTCCGCCTCSAG-3′ and reverse 5′-GATRTTCTTGCGCCAGTCTC-3′,
5. *anp* forward 5′-AGACCTGATGGATTTCAAGA-3′ and reverse 5′-GCTCCAATCCTGTCCATCCT-3′, and
6. *α-tubulin* forward 5′-CCACAGCTTTGGTGGGGGAA-3′ and reverse 5′-CATGGTAGGCTTTCTCAGCA-3′.

The R and S symbols indicate degenerate primers (R = A, G; S = C, G) and the same primers were used for cloning and RT-PCR experiments.

### PCR Parameters and Agarose Gel Electrophoresis

Each PCR reaction contained 5 μL of cDNA dilution, 1.25 μL of primer pair mix (0.3 nmol/μL each of forward and reverse primer), 2.5 μL of 10X PCR buffer (Invitrogen), 1.5 μL MgCl_2_ (50 mM), 0.5 μL dNTP (10 mM), 1 μL Taq polymerase (Invitrogen), and 13.25 μL DEPC-treated water. Amplification began with a 7 min incubation at 95°C followed by 30–35 cycles of 95°C for 1 min, annealing at an experimentally determined temperature for 1 min, and 72°C for 1 min. The process ended with 72°C for 10 min before returning to 10°C. Annealing temperatures were 68.6, 62.5, 53.8, 62.5, 53.8, and 53.0 for *gata4*, *nkx2–5*, *troponin C*, *troponin I*, *anp*, and *α-tubulin*, respectively. Xylene Blue loading dye (2 μg per PCR reaction) was added to all PCR products before separation on 1% agarose gels containing ethidium bromide at 130 V for 18 min. All dilutions were run for each gene and the most dilute cDNA samples that successfully amplified the gene of interest (10^-2^ or 10^-3^) were used for quantification.

### Sequencing of PCR Products

Following visual analysis on agarose gels, amplified PCR products were excised and frozen in liquid nitrogen for 5 min, thawed, and frozen/thawed again. After centrifugation at 12,000 rpm for 5 min, PCR products were forced through a 0.5 mL Eppendorf tube (with a hole punctured at the bottom with an 18 gauge syringe). The PCR products were forced through to another tube while the remaining agarose was filtered out by glass wool. Amplified cDNA was collected in a 1.5 mL tube. DNA was briefly vortexed with 0.1 volume of 3 M sodium acetate and 3 volumes of 90% isopropanol. Following a 15 min centrifugation at 12,000 rpm, the supernatant was discarded and the pellet was rinsed with 70% ethanol. Pellets were allowed to dry for 10 min before they were dissolved in 25 μL DEPC-treated water. Purified cDNA samples were sequenced by Bio Basics Inc. (Markham, Ontario) and results were analyzed in BLASTn at NCBI to confirm identity of the amplified gene. The *gata4*, *nkx2–5*, *troponin I*, *troponin C*, and *anp* products correspond to the GenBank accession numbers KC466328, KC466327, KC466326, KC466325, KC466329, respectively.

### Preparation of Total Soluble Protein Extracts

Total soluble protein preparations were prepared as previously described [25] and was separately extracted from the hearts of 4 animals for each of the six experimental stages. Briefly, frozen samples were quickly weighed, crushed into small pieces under liquid nitrogen and then homogenized (using a Polytron PT10) 1:3 w:v in ice-cold homogenizing buffer (20 mM HEPES, pH 7.5, 200 mM NaCl, 0.1 mM EDTA, 10 mM NaF, 1 mM Na_3_VO_4_, 10 mM β-glycerophosphate) with the addition of a few crystals of phenylmethylsulfonyl fluoride (PMSF) and 1 μl Protease Inhibitor Cocktail (BioShop; Cat. # PIC001). Samples were centrifuged at 10,000 rpm for 10 min at 4°C and supernatants were removed. Soluble protein concentration was assayed with the Bio-Rad protein reagent (BioRad Laboratories, Hercules, CA; Cat # 500–0006) and a MR5000 microplate reader at 595 nm. Final protein concentrations were adjusted to 10 μg/μL by the addition of a small volume of homogenizing buffer. Samples were then combined 1:1 v:v with 2X SDS sample buffer (100 mM Tris-base, 4% w/v SDS, 20% v/v glycerol, 0.2% w/v bromophenol blue, 10% v/v 2-mercaptoethanol, pH 6.8), boiled for 5 min and then stored at -40°C until use.

### Preparation of Nuclear Protein Extracts

Nuclear proteins extracts were prepared as previously described [25] and was separately extracted from the hearts of 4 animals for each of the six experimental stages. Frozen heart samples were homogenized 1:2 w:v using a Dounce homogenizer (20 piston strokes) in lysis buffer (10 mM HEPES, pH 7.9, 10 mM KCl, 10 mM EDTA, 20 mM β-glycerophosphate), with 10 μL of 100 mM DTT, 10 μL of protease inhibitor cocktail, and a few crystals of PMSF added immediately before homogenization. Samples were centrifuged for 10 min at 10,000 rpm at 4°C and supernatants were removed as the cytoplasmic fractions. Pellets were resuspended in 147 μL of nuclear extraction buffer (20 mM HEPES, pH 7.9, 400 mM NaCl, 1 mM EDTA, 10% v/v glycerol, 20 mM β-glycerophosphate) with 1.5 μL of 100 mM DTT, and 1.5 μL of protease inhibitor cocktail added. Samples were incubated on ice with gentle rocking for 1 h and then centrifuged for 10 min at 10,000 rpm at 4°C. Protein concentrations were determined with the Bio-Rad protein assay, adjusted to 5 μg/μL, and samples were stored at -80°C until use.

### Western Blotting

Total protein extracts were loaded onto 10% SDS-polyacrylamide gels and run at 180 V for 45 min as previously described [25]. For GATA4, p-GATA4, Nkx2–5, troponin C and troponin I, 20 μg of protein was loaded in each well. For ANP, 15% SDS-polyacrylamide gels were used loaded with 40 μg samples. Proteins were transferred to PVDF membranes by electroblotting at 160 mA for 90 min in transfer buffer (25 mM Tris-base, pH 8.5, 192 mM glycine, 10% v/v methanol). Membranes were then blocked with 3% w/v milk in TBST (20 mM Tris-base, pH 7.6, 140 mMNaCl, 0.05% v/v Tween-20) for 15 min. Membranes were probed with primary antibodies diluted 1:1000 v/v in TBST at 4°C overnight. Antibodies specific for mammalian GATA4 (Cat no. sc-9053, human origin), p-GATA4 (detects phosphorylated Ser 262 in human, which is equivalent to Ser 261 in mouse and rat) (Cat No. sc-32823, human origin), Nkx2–5 (Cat No. sc-14033, human origin) and troponin C (Cat No. sc-20642, human origin) were purchased from Santa Cruz Biotechnology. Troponin I antibody (Cat No. TI-1, rabbit origin) was from the University of Iowa Developmental Studies Hybridoma Bank and ANP antibody (Cat No. GTX109255, human origin) was purchased from GeneTex. Antibodies cross-reacted with single bands on the immunoblots at the expected molecular weights of 45, 40, 18, 24 and 17 kDa for GATA4, Nkx2–5, troponin C, troponin I and ANP, respectively. Membranes were washed three times with TBST and then probed with HRP-linked anti-rabbit IgG secondary antibody (1:4000 v:v), except for anti-troponin I antibody, which was incubated with HRP-linked anti-mouse IgG secondary antibody (1:2000 v:v). After a second set of washings, bands were visualized by enhanced chemiluminescence (H_2_O_2_ and luminol) and band densities were quantified. Subsequently, blots were stained with Coomassie blue (0.25% w/v Coomassie brilliant blue, 7.5% v/v acetic acid, 50% methanol) to visualize all protein bands.

### Transcription Factor-DNA Binding ELISA

DNA oligonucleotides were designed based on the DNA binding elements of GATA4 and Nkx2–5 transcription factors and produced by Sigma Genosys. The biotinylated probe (GATA4 5’-Biotin-GCCTAAGCCAAGTGATAAGCAGCCAGACAA-3’ and Nkx2–5 5’-Biotin-CCACTCAAGTGCACTCAAGT-3’) and the complement probe (GATA4 5’-Biotin-TTGTCTTGGCTGCTTATCACTTGGCTTAGGC-3’ and Nkx2–5 5’-Biotin-ACTTGAGTGCACTTGAGTGG-3’) were first diluted in sterile water (500 pmol/μl) and mixed 1:1 v:v for a total of 20 μl. Probes were then placed in a thermocycler for 10 min at 9500B0030C and slowly cooled to room temperature. Double stranded probes were diluted in phosphate buffered saline (PBS; 137 mM NaCl, 2.7 mM KCl, 10 mM Na_2_HPO_4_, 2 mM KHPO_4_, pH 7.4) and 50 μL of diluted DNA probe was added (40 pmol DNA/well) to streptavidin coated wells on a microplate. Following a 1 h incubation, unbound probe was discarded and wells were rinsed three times in wash buffer (1X PBS containing 0.1% Tween-20), and a fourth time with 1X PBS. Equal amounts of protein from nuclear extracts were vortexed with transcription factor binding buffer (10 mM HEPES, 50 mM KCl, 0.5 mM EDTA, 3 mM MgCl_2_, 10% v/v glycerol, 0.5 mg/ml bovine serum albumin, 0.05% NP-40, 20 mM DTT, pH 7.9). In each well containing DNA probe, 50 μL of the nuclear protein preparation was added, with the exception of negative control wells which contained transcription factor binding buffer without protein. Following 1 h incubation with gentle shaking, protein mixtures were discarded and the wells were washed three times with wash buffer and once with 1X PBS.

Diluted primary antibody was then added (60 μL/well) and allowed to incubate for 1 h. Excess antibody was then discarded, and wells were rinsed three times with wash buffer and once with 1X PBS before incubation with diluted secondary antibody (60 μL/well) for 1 h. This antibody was then discarded and wells were rinsed four times with wash buffer. Primary and secondary antibodies were the same as those used for Western Blots. All quantitative runs contained 50 μg of protein/well, 1 μg (GATA4) or 2 μg (all others) of salmon sperm/well, 30 mM NaCl (or 50 mM for Nkx2–5), 1:2000 v:v anti-rabbit secondary antibody in TBST, and one of the primary antibodies in TBST: GATA4 (1:1000 v:v), p-GATA4 (1:2000 v:v), or Nkx2–5 2 (1:1000 v:v). After secondary antibody incubation and washing, bound antibody was detected using tetramethylbenzidine (TMB) (BioShop). A 60 μL aliquot of TMB was added to each well, colour was developed, and then the reaction was stopped with 60 μL of 1 M HCl. Absorbance was measured at 450 nm (reference wavelength of 655 nm) using a Multiskan spectrophotometer.

### Quantification and Statistics

Band intensities of ethidium bromide-stained agarose gels and chemiluminescent immunoblots were visualized using a Chemi-Genius BioImaging system (Syngene, Frederick, MD) and quantified with the accompanying GeneTools software. Band intensities for PCR products of target genes were standardized against *α-tubulin* amplified from the same cDNA sample. Band intensities from immunoblots were standardized against the total intensity of a group of Coomassie stained protein bands from the same sample; all samples (control and experimental) were also previously shown to have constant expression of α-tubulin as determined from both immunoreactive band densities alone and also when standardized against the Coomassie stained bands. Data are expressed as means ± SEM, n = 4–5 biological replicates. Statistically significant differences (p < 0.05) between the experimental groups were determined using one-way ANOVA with Tukey’s post-hoc test using SigmaPlot 11 software (Systat Software Inc., San Jose, CA).

## Results

### *Gata4* and *nkx2–5* Transcript Levels

**Fig 1:**
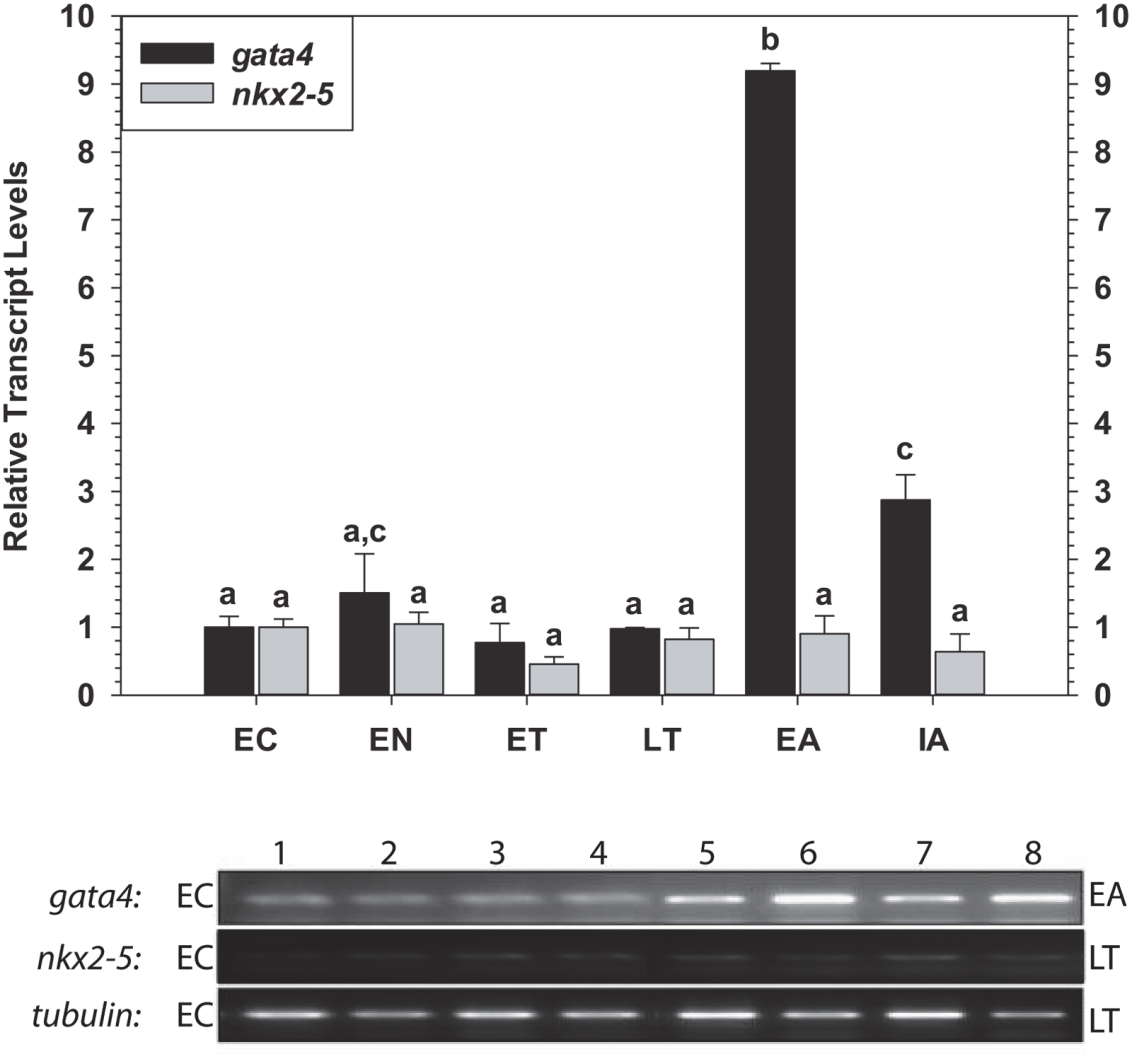
Relative changes in mRNA transcript levels of *gata4* and *nkx2–5* in the heart of *I. tridecemlineatus* over the torpor-arousal cycle. Sampling points were: euthermic in the cold room (EC), entrance into torpor (EN), early torpor (ET), late torpor (LT), early arousal (EA), and interbout arousal (IA); see Materials and Methods for more extensive explanations. Representative bands on agarose gels are shown for selected pairs of sampling points that are labeled to the left (lanes 1–4) and right (lanes 5–8) of the gel. Transcript levels for each gene were standardized against band densities for *α-tubulin* amplified from the same sample. Relative transcript levels are shown in histograms expressed as mean standardized band densities (± S.E.M., n = 4 independent RNA isolations from different animals) for each of the six sampling points. Data were analyzed using analysis of variance with a post hoc Tukey test (p<0.05); for each gene, values that are not statistically different from each other share the same letter notation.

RT-PCR was used to quantify relative changes in *gata4* and *nkx2–5* transcript levels in ground squirrel heart over the torpor-arousal cycle (Fig. 1). Transcript levels of *gata4* remained constant throughout entry into torpor (EN) and during both early (ET) and late (LT) stages of torpor. However, *gata4* transcripts increased strongly and significantly by 9.2-fold during early arousal (EA) as compared to euthermic controls (EC, P<0.001). Transcript levels decreased again in animals sampled after ~18 h of interbout arousal (IA), but still remained significantly elevated by 2.9-fold with respect to EC (P = 0.004). By contrast, relative transcript expression of *nkx2–5* remained constant over the torpor-arousal cycle.

### GATA4, p-GATA4 and Nkx2–5 Protein Levels

**Fig 2:**
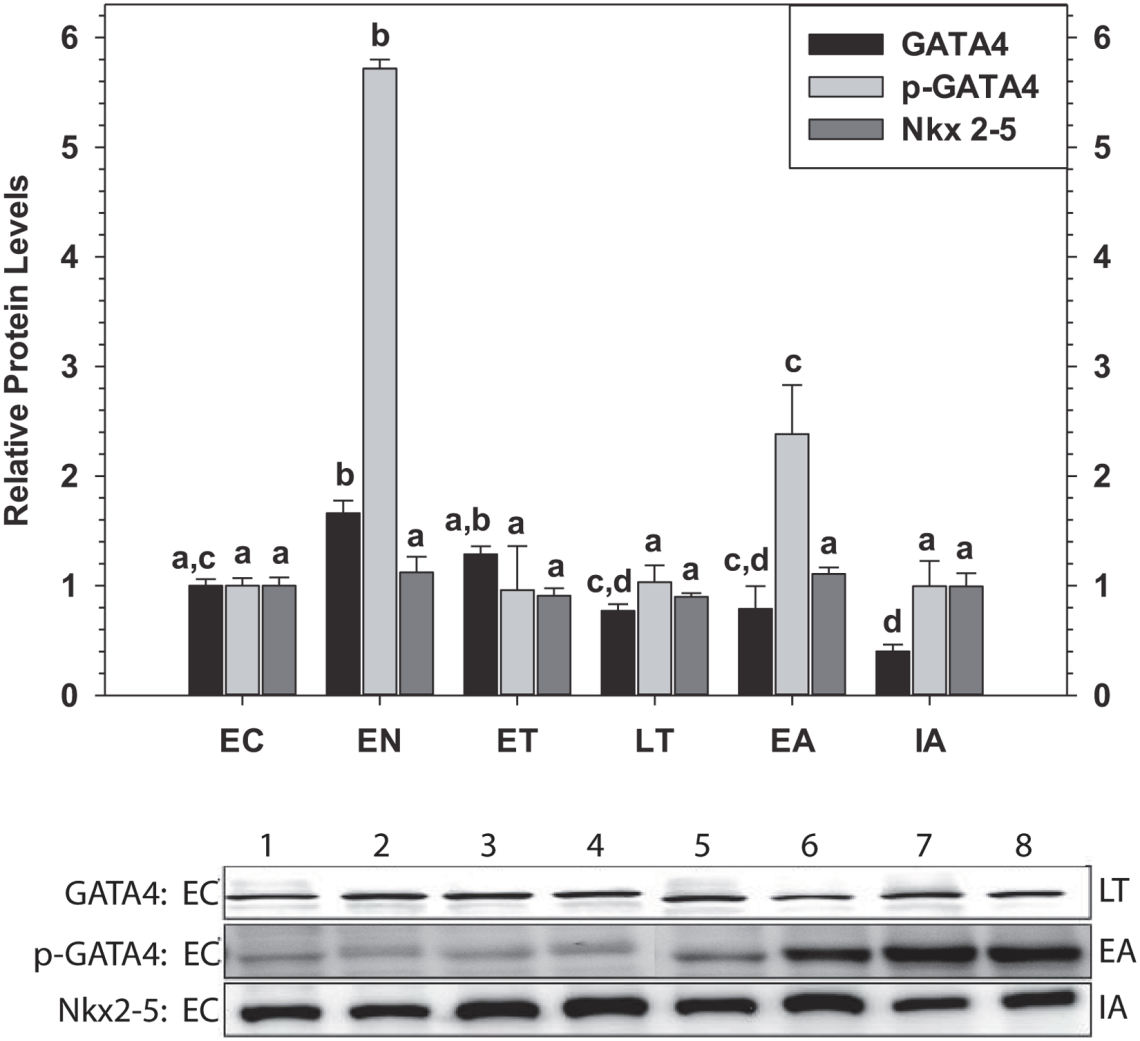
Relative changes in the protein levels of the transcription factors GATA4 and Nkx2–5 as well as the phosphorylation state of GATA4 (Ser 261) in the heart of *I. tridecemlineatus* over the torpor-arousal cycle. Representative Western blots are shown for selected pairs of sampling points that are labeled to the left (lanes 1–4) and right (lanes 5–8) of the gel. Histograms show mean standardized band densities (± S.E.M., n = 4 independent protein isolations from different animals). Other information as in Fig. 1.

Immunoblotting was use to assess changes in the relative protein levels of GATA4 and Nkx2–5 in ground squirrel heart over the torpor-arousal cycle as well as the phosphorylation state of GATA4 at Ser 261 (Fig. 2). Phosphorylation of GATA4 at Ser 261 has been previously established as a marker of transcription factor activity [30–31]. As compared to EC, total protein levels of GATA4 increased significantly by 1.7-fold in EN (P = 0.005), before returning to EC levels during ET, LT, and EA. During interbout arousal, however, total GATA4 protein levels decreased significantly to just 40% of EC values (P = 0.012). Similar to the GATA4 protein expression pattern, phosphorylation of GATA4 at Ser 261 also increased significantly in EN, rising strongly to levels that were 5.7-fold higher than EC (P<0.001). Phosphorylation state was reduced during torpor but peaked again during EA to 2.4-fold higher than EC (P = 0.023). Total protein levels of Nkx2–5 did not change significantly over the torpor-arousal cycle.

### GATA4, p-GATA4 and Nkx2–5 Relative Binding to DNA

**Fig 3:**
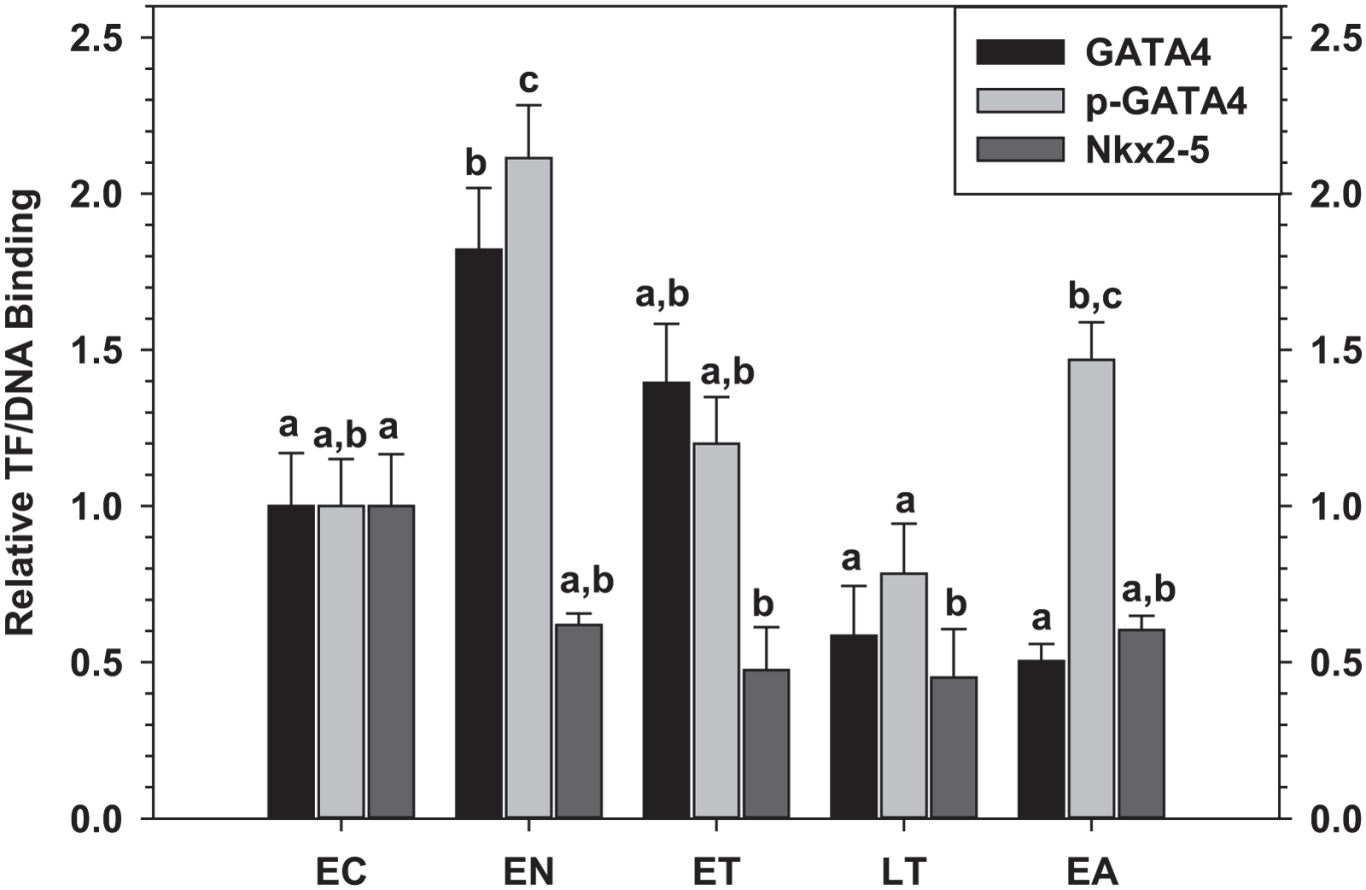
Relative changes in binding of the transcription factors GATA4 and Nkx2–5 as well as the phosphorylated form of GATA4 (Ser 261) to DNA binding elements in the heart of *I. tridecemlineatus* over the torpor-arousal cycle. Optical density readings were corrected by subtraction of blanks containing no protein and values were expressed relative to EC. Histograms show mean relative values ± S.E.M., n = 4 independent biological replicates for each of the five experimental conditions. Other information as in Fig. 1.

A modified enzyme-linked immunosorbent assay (ELISA) was used to study the interactions of transcription factors in nuclear extracts of squirrel heart with DNA oligonucleotides containing the target promoter regions of the transcription factors GATA4 and Nkx2–5 (Fig. 3). Relative binding to DNA was measured for five time points: EC, EN, ET, LT, and EA. GATA4 binding to its target promoter element was significantly higher by 1.8-fold during EN as compared to EC (P = 0.02). GATA4 binding then began to decrease in ET towards levels similar to EC, and was significantly decreased below EN values during LT and EA; for example, compared to peak values in EN, values during LT and EA were just 32% (P<0.001) and 27% (P<0.001), respectively. A similar pattern was observed when the anti-p-GATA4 primary antibody (detects p-Ser 261) was used to study the binding by phosphorylated GATA4 to DNA. Binding by p-GATA4 also increased significantly by 2.1-fold in EN as compared to EC (P = 0.001), and then fell during ET and LT to levels similar to EC. However, in contrast to the pattern detected with the total GATA4 antibody, p-GATA4 binding to DNA increased significantly in EA by 1.9-fold as compared to LT (P = 0.048). Relative binding of Nkx2–5 remained constant throughout the torpor-arousal cycle with the exception of ET and LT, where relative binding was significantly reduced to 47% (P = 0.05) and 45% (P = 0.04) of EC values, respectively.

### *Troponin I*, *troponin C* and *anp* Transcript Levels

**Fig 4:**
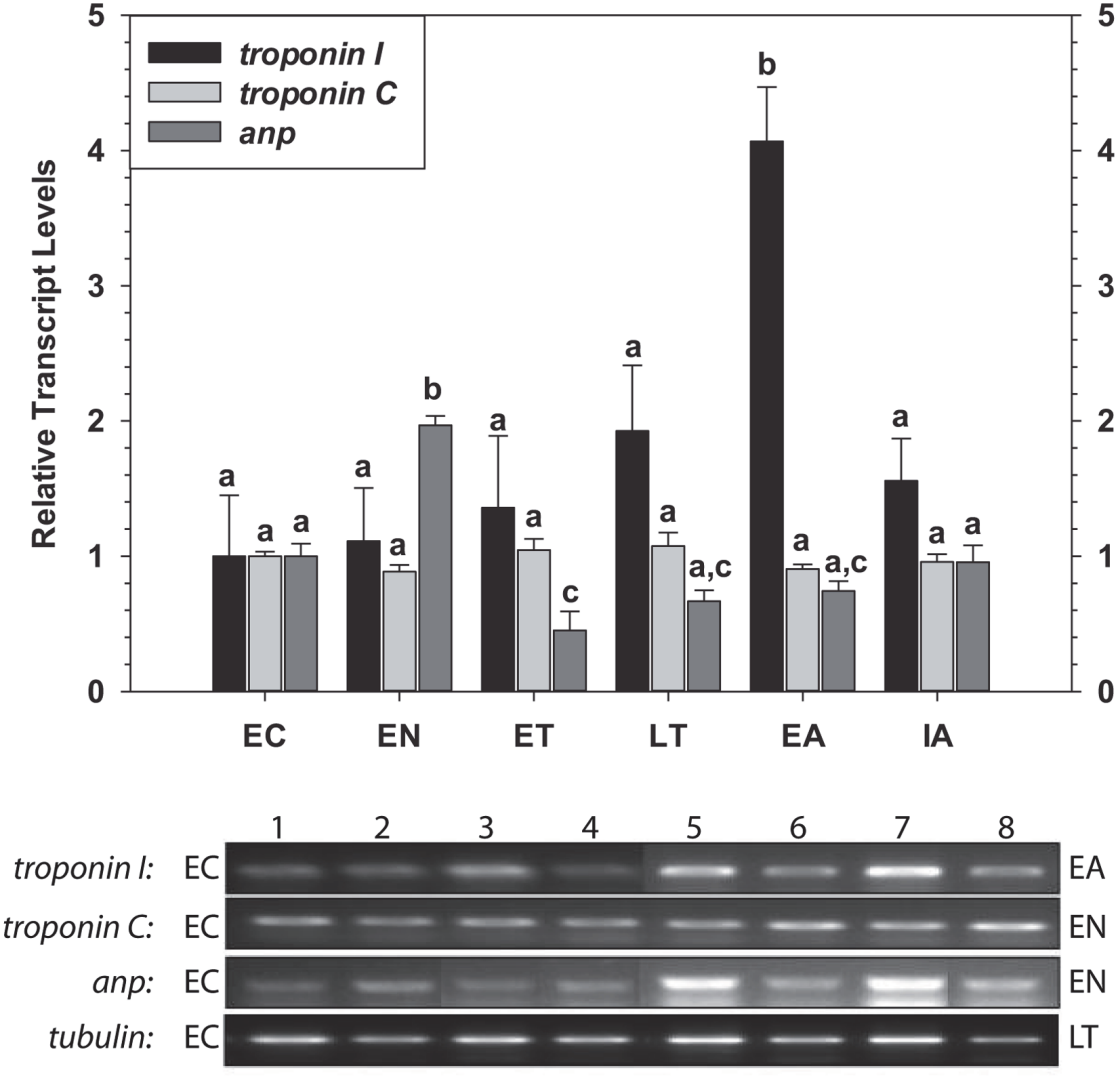
Relative changes in mRNA transcript levels for *troponin I*, *troponin C* and *anp* in the heart of *I. tridecemlineatus* over the torpor-arousal cycle. Other information as in Fig. 1.

Changes in the transcript levels of three GATA4 downstream genes, *troponin I*, *troponin C* and *anp*, were also assessed over the torpor-arousal cycle (Fig. 4). While Troponin T is also part of the troponin complex, it was not measured in the present study since MEF2 is thought to be the primary regulator, although MEF2 and GATA4 may both play a role. Transcript levels of *troponin I* remained constant over the torpor-arousal cycle with the exception of EA when levels increased greatly by 4.1-fold, as compared to EC (P<0.001). Transcript levels of *troponin C* did not change significantly throughout the torpor-arousal cycle. However, transcript levels of *anp* rose significantly by 2.0-fold during EN (P<0.001) but decreased below control values during ET (to 45% of EC, P = 0.009 or 23% of EN, P<0.001).

### Troponin I, Troponin C and ANP Protein Levels

**Fig 5:**
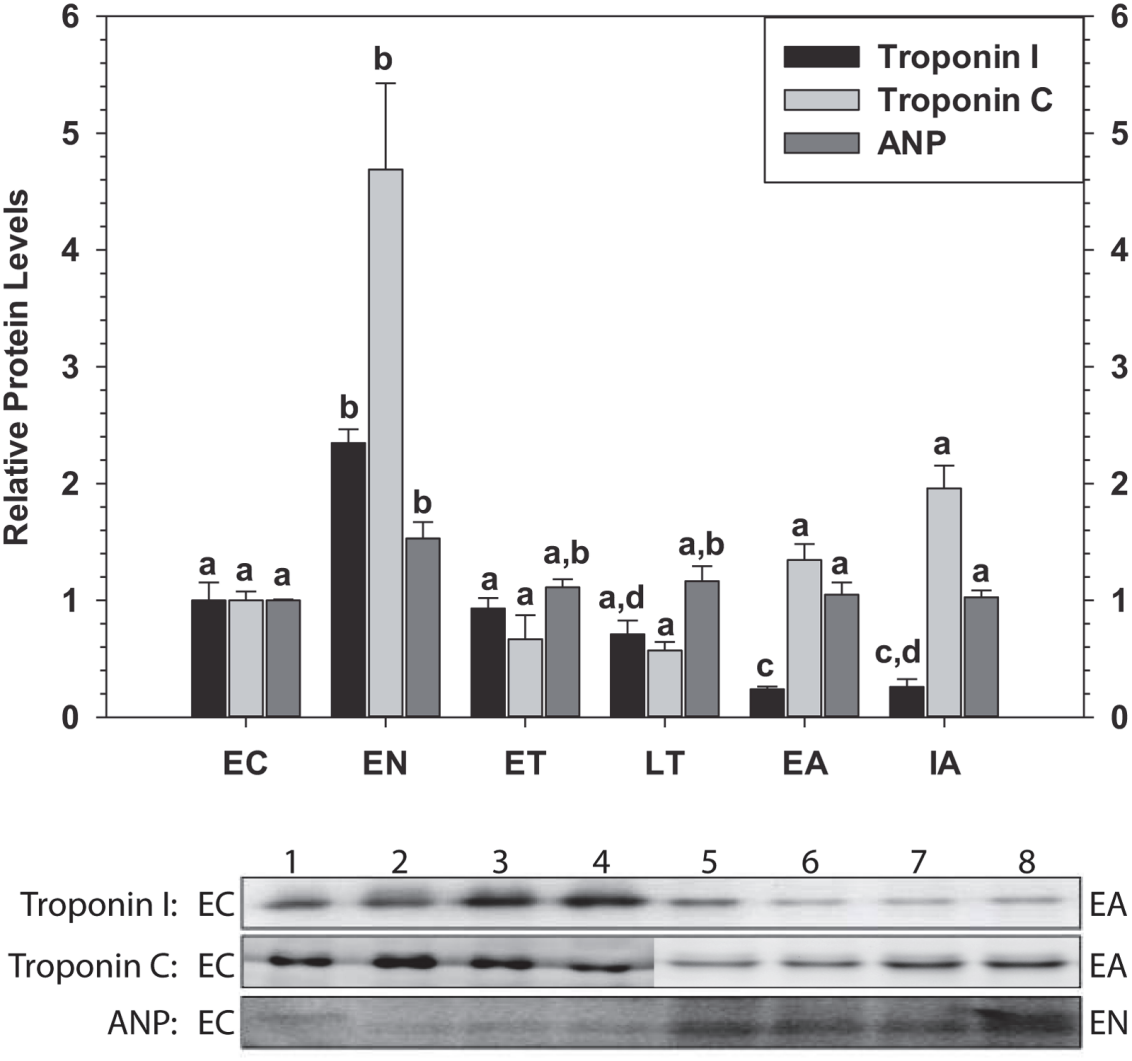
Relative changes in the protein levels of Troponin I, troponin C, and ANP in the heart of *I. tridecemlineatus* over the torpor-arousal cycle. Other information as in Fig. 2.

Troponin I protein levels increased significantly by 2.3-fold in heart during EN when compared to EC (P<0.001, Fig. 5). Levels then declined to values similar to EC during torpor (both ET and LT), but decreased strongly during arousal to levels that were just 24% (P<0.001) and 26% (P = 0.001) in EA and IA, respectively, of the corresponding EC values. Relative protein levels of troponin C also increased significantly in EN (P<0.001), reaching 4.7-fold higher than EC, but then decreased again to near EC values over the remainder of the torpor-arousal cycle. ANP protein levels also increased significantly during EN (1.5-fold over EC, P = 0.012) but were not significantly different from EC values over the remainder of the torpor/arousal course.

## Discussion

The goal of this study was to explore molecular adaptations of cardiac muscle to the stresses experienced during hibernation using a well-researched model hibernator, the thirteen-lined ground squirrel. Recently, we have shown evidence that MEF2A and MEF2C play key roles in preparing cardiac muscle for cycles of torpor-arousal [22]. In the present study, we hypothesized that transcription factors (GATA4 and Nkx2–5) that have been well characterized in cardiomyogenesis, cardioprotection, and cardiomyocyte hypertrophy may also play key roles in molecular remodeling of the heart during hibernation. These transcription factors have been previously shown to regulate the expression of *troponin I*, *troponin C*, and *anp* [32–33, 50–52]; thus, we also characterized the gene/protein expression of these three downstream targets over the torpor-arousal cycle.

Troponin I is a structural protein in the troponin complex which prevents the interaction between myosin and actin by inhibiting actomyosin ATPase, resulting in inhibition of muscle contraction [41–43]. Furthermore, it has been well-studied as a biomarker to identify cardiac stress or damage in clinical applications [39–41]. Stress and damage to heart tissue have been shown to cause modifications to troponin I, which ultimately hamper cardiac muscle contractibility, decrease maximum tension and thick filament sliding speed, and actin-myosin affinity [53–54]. This led us to hypothesize that troponin I may be an important target which facilitates the active decrease in heart rate seen during entrance into torpor and/or contributes to survival strategies which allow the heart to sustain lower temperatures, reduced blood flow, and altered energy metabolism during torpor. Furthermore, the regulation of troponin I may also be critical during interbout arousal whereby an increase in heart rate is observed and the torpid phenotype is rapidly reversed. Indeed, our data showed increased protein expression of troponin I only during EN, a return to normal during sustained torpor, and a fall to low levels during interbout, suggesting the regulation of troponin I levels may be important during both cooling and rewarming. A study of rainbow trout ventricle revealed that cold acclimation resulted in an increase in cardiac troponin I expression, which complements their previous study that demonstrated low temperature-induced trout cardiac hypertrophy [55–56]. As such, one theory is that increases in troponin I may remodel the heart contractile machinery in response to temperature decrease which would allow a smooth transition into torpor to occur without the cardiac arrest that would occur if a human heart was cooled. Although a promising hypothesis, a clear discrepancy was observed between troponin I protein and mRNA data; protein was minimal with elevated transcript levels during arousal and, similarly, low transcript levels occurred with high protein levels during entrance. As torpor-arousal is cyclical in nature, it may be proposed that increased transcript expression during arousal reflects a preparatory response, occurring when body temperatures and metabolic rate are most conducive to active mRNA synthesis. In turn, *troponin I* may be rapidly translated in each successive entrance phase in order to facilitate transitions to cold body temperatures (Fig. 5).

Troponin C is another protein in the troponin complex that facilitates the interaction between myosin and actin when triggered by Ca^2+^ions, which ultimately results in muscle contraction. Troponin C sensitivity allows for detection of small shifts in calcium ion concentration which ultimately influence heart contractile performance and current research suggests that troponin C plays a role in sensing physiological and pathological stimuli [57]. Furthermore, studies in hibernators have found that the low body temperatures experienced during hibernation decreases calcium sensitivity of cardiac myofilaments [11] and ground squirrel hearts have the capacity to regulate calcium ion homeostasis despite the challenges associated with cold torpor, as compared to non-hibernating mammals [10, 59]. Taken together, the differential expression of troponin C in thirteen-lined ground squirrel heart during EN may be part of an adaptive mechanism that regulates calcium ion homeostasis and modifies the contractile machinery to deal with the necessary changes in heart function during cooling (Fig. 5). This theory is further complemented by another study which has shown that cold-tolerant teleosts produce more cardiac troponin C when acclimated to colder temperatures [58]. Similarly, the temperature-dependent troponin C expression and increased Ca^2+^ affinity in teleost cardiac troponin C has also been proposed to be an adaptive mechanism to allow for proper contraction at lower temperatures [58]. Indeed, our data, along with data observed in teleosts [58], would support the hypothesis that increases in troponin C expression may occur in other cold-adapted animals and, by extension, may represent a generalized adaptive mechanism which preserves cardiomyocyte function at cold temperatures. Although both troponin I and C protein levels increase during EN, troponin C transcript levels were not regulated similarly to troponin I levels (Figs. 4 and 5). This suggests that both troponins may be subject to intricate posttranscriptional regulatory programs and further highlights the complexities of the adaptive mechanisms of the heart. In summary, the striking similarities shared between the hibernating thirteen-lined ground squirrel and other cold-acclimated model organisms attests that the squirrel is an ideal mammalian model to study the molecular mechanisms of low-temperature biology as it pertains to cardiac muscle function and that constituents of the troponin complex are integral to this response.

ANP has been widely studied as a physiological regulator. Current knowledge suggests that ANP is an anti-hypertensive hormone and down-regulation of ANP in humans causes cardiac hypertension [47]. For cells under hypoxic stress, ANP plays a role in relaxing constricted pulmonary vessels, lowering pulmonary arterial pressure, and hampering both stress-induced pulmonary hypertension and cardiac remodelling [45–46]. In mouse models of dilated cardiomyopathy, ANP was found to reduce the chances of heart failure, pathological remodelling and mortality, while maintaining normal systolic function [60]. A study on salmon cardiac peptides (sCP), which are analogous to ANP and brain natriuretic peptide (BNP) in mammals, found that sCP mRNA increased at lower temperatures, coinciding with heart hypertrophy [61]. Similar to salmon, we hypothesize from our results that the hibernating thirteen-lined ground squirrel may be relying on ANP to achieve versatile regulation of heart pressure, physiology, and endocrinology. ANP may be regulating cardiac processes to promote optimal heart function during the transition into torpor and support the squirrel’s survival throughout the torpor-arousal cycle. In support of this, we found that both *anp* transcript and ANP protein levels increased during EN, with respect to EC (Figs. 4 and 5) but returned to control levels over the rest of the torpor-arousal cycle. One can further hypothesize that the role of ANP during hibernation is to regulate fluid dynamics via whole body blood pressure changes. Although biologists have understood that natriuretic peptides play a role in regulation of salt-water balance, major advancements in their physiological mechanism are still emerging [62]. In theory, ANP expression during hibernation can be beneficial to the animal as vasoconstriction leads to water loss via the kidneys. Expressing ANP may override vasoconstriction that occurs in response to cold, which is valuable particularly in winter, with sparse water availability. Therefore, the differential expression of ANP seen in this study may benefit the hibernator by regulating both blood pressure and salt-water homeostasis.

The transcription factors GATA4 and Nkx2–5 are crucial to development of the heart. Both transcription factors have been extensively characterized as regulators of the genes of current interest (troponin C, troponin I and ANP), but mutual dependence on one another for gene expression has also been demonstrated [32–38]. Analysis of *gata4* and *Nkx2–5* mRNA levels showed that *gata4* transcripts were significantly up-regulated during arousal from torpor whereas *nkx2–5* transcript levels were unchanged over the torpor-arousal cycle (Fig. 1). However, GATA4 protein levels only increased during EN (Fig. 2). As suggested above for troponin I, *gata4* transcripts may be increased during arousal as a preparatory measure to support elevated translation during the next entrance phase into torpor. Levels of phosphorylated GATA4, a modification previously linked to transcriptional activation [30–31], were greatest in EN and also elevated in EA suggesting that GATA4 mediated gene expression is most active during these two phases of the torpor-arousal cycle (Fig. 2). Complimentary to this, GATA4 and p-GATA4 binding to the target element increased strongly during EN as compared to EC and p-GATA4 binding increased moderately during EA, as compared to LT values (Fig. 3). Increased activation of GATA4 during EN and EA was correlated with increases in *anp* and *troponin I* transcript levels, respectively, suggesting that GATA4 regulates the expression of these genes at distinct time points during the torpor-arousal cycle. Although Nkx2–5 DNA binding either remained constant or decreased, analysis of crosstalk between Nkx2–5 and GATA4 will require further studies. Since both GATA4 and Nkx2–5 are required for ANP transcription, the increase in GATA4 activity alone may be sufficient to explain the increases in ANP mRNA levels since Nkx2–5 is still present in nuclear extracts to act as its partner (Fig. 2) [32–33]. This conclusion is in agreement with a recent study that demonstrates that GATA4, unlike Nkx2–5, may have important roles in the biochemical regulation of the postnatal heart [63]. Alternatively, despite decreased Nkx2–5 total protein, transcriptional activity has been demonstrated to improve with SUMOylation, suggesting that a survey of post-translational modifications associated with Nkx2–5 activity may provide more information about its role during hibernation [64]. In summary, our results suggest that GATA4 regulation begins at the arousal stage, but also acts to ensure expression of key target genes during entrance (EN) into the next period of torpor. Ultimately, the differential regulation of GATA4 and its downstream genes may facilitate important molecular and biochemical changes in the heart over cycles of torpor-arousal. Considering the prominent role of GATA4 in the thirteen-lined ground squirrel, and the results of previous studies which have shown the cardioprotective roles played by GATA4, further studies should examine the regulation of erythropoietin, apoptosis, and oxidative stress pathways as adaptive mechanisms in hibernation [26–27].

## Conclusions

Our findings demonstrate that GATA4 and its downstream targets are regulated in the heart of the hibernating thirteen-lined ground squirrel over the torpor-arousal cycle with significant changes occurring particularly in entrance and early arousal stages. Nkx2–5, the binding partner of GATA4, is not differentially regulated over the torpor-arousal cycle, but is nevertheless present and capable of interacting with GATA4 to facilitate increased expression of *troponin I*, *troponin C*, and *anp*. The transcription, translation, and post-translational modification of GATA4 are regulated throughout the torpor-arousal cycle and this regulation is essential to achieving increases in the relative expression of downstream genes. In turn, changes in the relative expression levels of Troponin I, Troponin C, and ANP may represent adaptive mechanisms which will facilitate the expected physiological changes such as shifts in heart contraction rate and coping mechanisms to deal with changes in blood pressure.
